# Unconventional Approach: Steroid Treatment for Eosinophilic Myocarditis Despite Negative Endomyocardial Biopsy

**DOI:** 10.7759/cureus.40845

**Published:** 2023-06-23

**Authors:** Michael Vaysblat, Suhwoo Bae, Beatrice Panjwani, Michael Esposito, Calvin Ngai, Matthew Pierce

**Affiliations:** 1 Medicine, North Shore University Hospital (NSUH), Brooklyn, USA; 2 Internal Medicine, Zucker School of Medicine at Hofstra/Northwell Residency Program, Manhasset, USA; 3 Radiology, Northwell Health, Manhasset, USA; 4 Pathology, Northwell Health, New Hyde Park, USA; 5 Cardiology, Northwell Health, Manhasset, USA

**Keywords:** fulminant necrotizing eosinophilic myocarditis, hyper-eosinophilic syndrome, hyper-eosinophilia, cardiac imaging-mri, focal myocarditis

## Abstract

Eosinophilic myocarditis (EM) is a rare type of myocarditis that can present acutely with rapidly progressing symptoms leading to high rates of morbidity and mortality. EM is defined by eosinophilic infiltration of the myocardium and can be difficult to diagnose even with gold-standard techniques, such as endomyocardial biopsy (EMB), given the possibility of patchy myocardial infiltration. Here, we present a case of idiopathic EM complicated by a cardiac arrest that was empirically treated with high-dose intravenous steroids after negative EMB. The patient’s symptoms and cardiac function significantly improved after treatment. This case highlights the ambiguity of certain presentations of EM, its complications, and the importance of empiric treatment to avoid poor outcomes.

## Introduction

Eosinophilic myocarditis (EM) is a myocardial disease characterized by eosinophilic infiltration of the myocardium. Acute cases of EM generally involve rapidly progressive necrosis of the myocardium, leading to rapid deterioration in cardiac function within several weeks, culminating in high mortality rates [1]. Chronic cases are usually secondary to systemic diseases, such as hypereosinophilic syndrome (HES), and usually lead to progressive symptoms of congestive heart failure over several years [1]. Clinical manifestations include a wide range of symptoms encompassing congestive heart failure and the triggering etiology such as parasitic infection or medication. These can include chest discomfort, dyspnea, lower extremity edema, fevers, rash, and joint pain [2]. The diagnosis of EM may prove challenging, especially in patients with atypical presentations. While endomyocardial biopsy (EMB) has long been considered the gold standard diagnostic modality for EM, there are several drawbacks. EMB can be deemed too invasive for some patients, which can limit its usage. The sensitivity of EMB is also not ideal, as EMB can present with focal infiltrates that can be missed even with experienced proceduralists. Cardiac MRI (CMR) has emerged as a formidable diagnostic tool that can aid in diagnosis, although it cannot definitively diagnose EM.

## Case presentation

**Figure 1:**
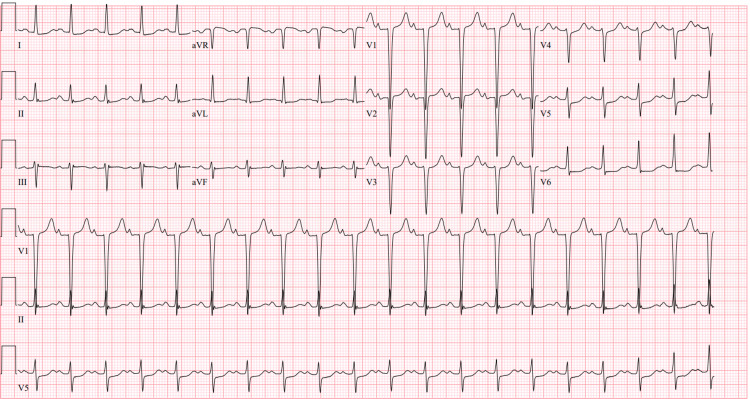
EKG on initial presentation was remarkable for sinus tachycardia of 118 bpm; otherwise, no ischemic changes, no ST-segment elevations or depressions, and no T wave abnormalities were noted

**Figure 2:**
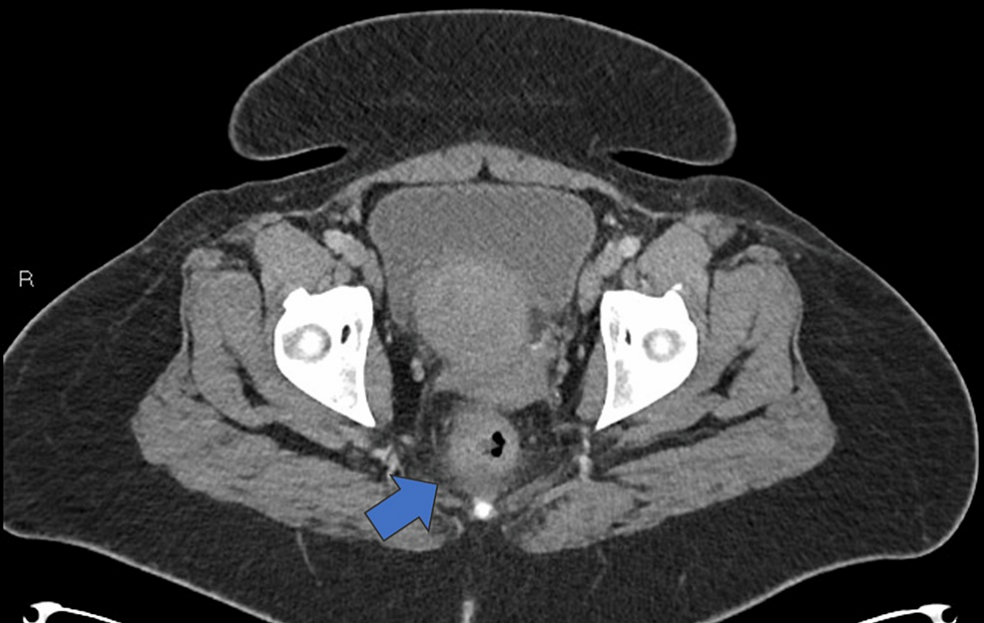
CT of the abdomen and pelvis on admission showing proctocolitis in the axial view (blue arrow)

**Figure 3:**
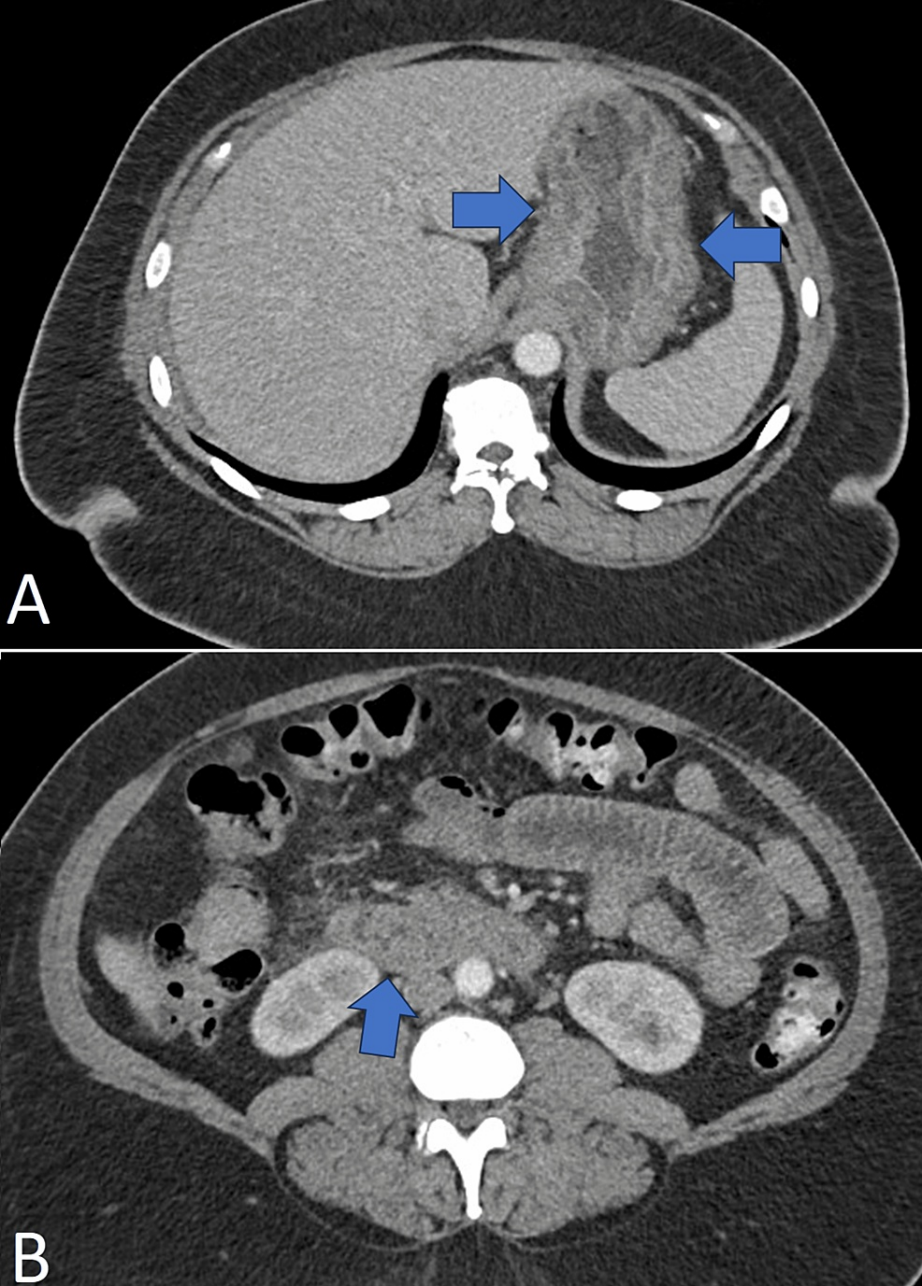
Repeat CT abdomen and pelvis Repeat CT of the abdomen and pelvis on hospital Day 4 showing (A) gastritis (blue arrows) and (B) duodenitis (blue arrow)

A 43-year-old female with a past medical history of nonischemic cardiomyopathy, type 2 diabetes mellitus, and hypertension was admitted with nausea, non-bilious vomiting, and left-sided abdominal pain. On initial vital signs, she had a fever of 100.4 degrees F, heart rate of 117 beats per minute, blood pressure of 186/145 mmHg, respiratory rate of 17 breaths per minute, and oxygen saturation of 100% on room air. The physical exam was unremarkable, with no tenderness to palpation in the abdomen. Initial labs were remarkable for an elevated white blood cell count (WBC), eosinophil count, and eosinophil percentage (Table 1). The electrocardiogram on admission revealed sinus tachycardia of 118 with no ST segment changes or T wave abnormalities (Figure 1). CT of the abdomen and pelvis with intravenous contrast was remarkable for proctocolitis, an indeterminate left adnexal mass, and an appendicolith measuring 0.9 cm with no signs of acute appendicitis (Figure 2). She was empirically started on intravenous piperacillin/tazobactam. Gastroenterology and infectious disease were consulted and recommended continuing current management. On hospital Day 4, a repeat CT abdomen and pelvis with intravenous contrast revealed gastritis and duodenitis (Figure 3). Ultrasound of the pelvis further characterized the left adnexal mass as a pedunculated leiomyoma, later confirmed by MRI pelvis. Transthoracic echocardiography was performed on hospital Day 5, which was significant for a left ventricular ejection fraction (LVEF) of 40-45% and moderate global left ventricular systolic dysfunction.

**Table 1:** Laboratory investigations during hospitalization IgE: immunoglobulin E

Laboratory Test	Initial Presentation	Mid Hospital Course	Diagnostic Workup	After Steroid Therapy	Reference Range
White blood cell (K/uL)	20.16	17.76		10.08	3.8 - 10.5
Eosinophil count (K/uL)	4.64			0.25	0 - 0.5
Eosinophil percentage (%)	23	19.9		2.5	0 - 6
Hemoglobin (g/dL)	13.6				11.5 - 15.5
Platelets (K/uL)	456				150 - 400
Alkaline phosphatase (U/L)	79				40 - 120
Aspartate aminotransferase (U/L)	12				15 - 37
Alanine transaminase (U/L)	17				12 - 78
Procalcitonin (ng/mL)		4.95			0.02 - 0.10
IgE level (KU/L)		66	58		<100
Homocysteine (umol/L)			10.8		<15
Factor V assay (%)			99		50 - 150
Factor VIII assay (%)			84		60 - 125
Protein S assay (%)			85		63 - 140
Protein C assay (%)			122		74 - 150
Tryptase (ug/L)			5.3		2.2 - 13.2

**Figure 4:**
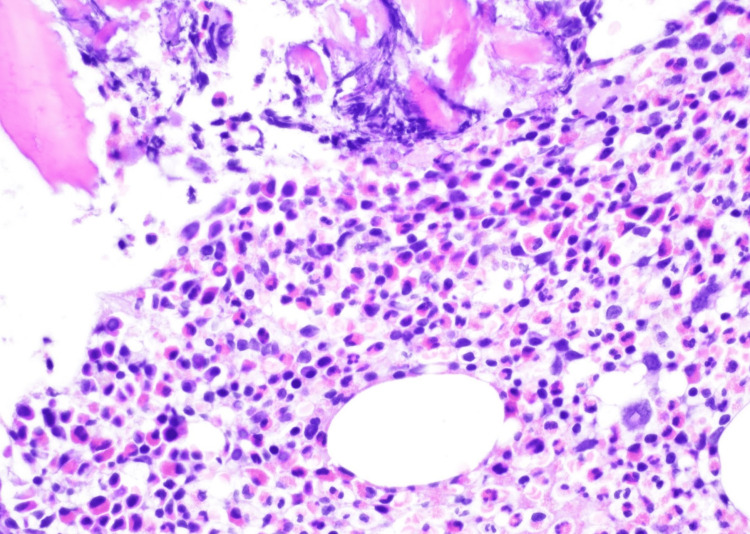
H&E staining of bone marrow biopsy remarkable for hypereosinophilia

**Figure 5:**
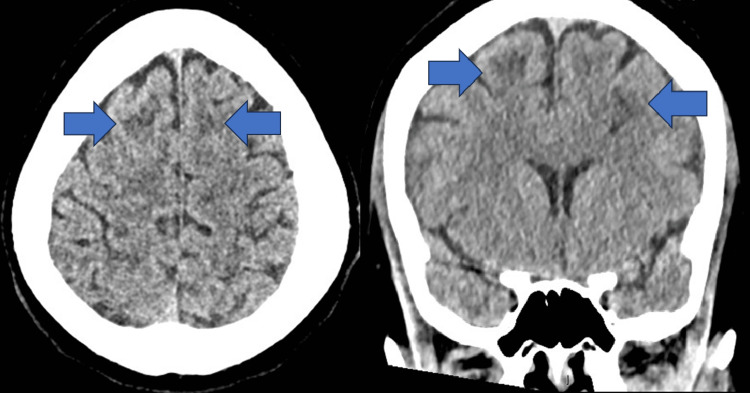
CT head without contrast showing focal hypodensities (blue arrows) apparent in both axial (left image) and coronal (right image) views

**Figure 6:**
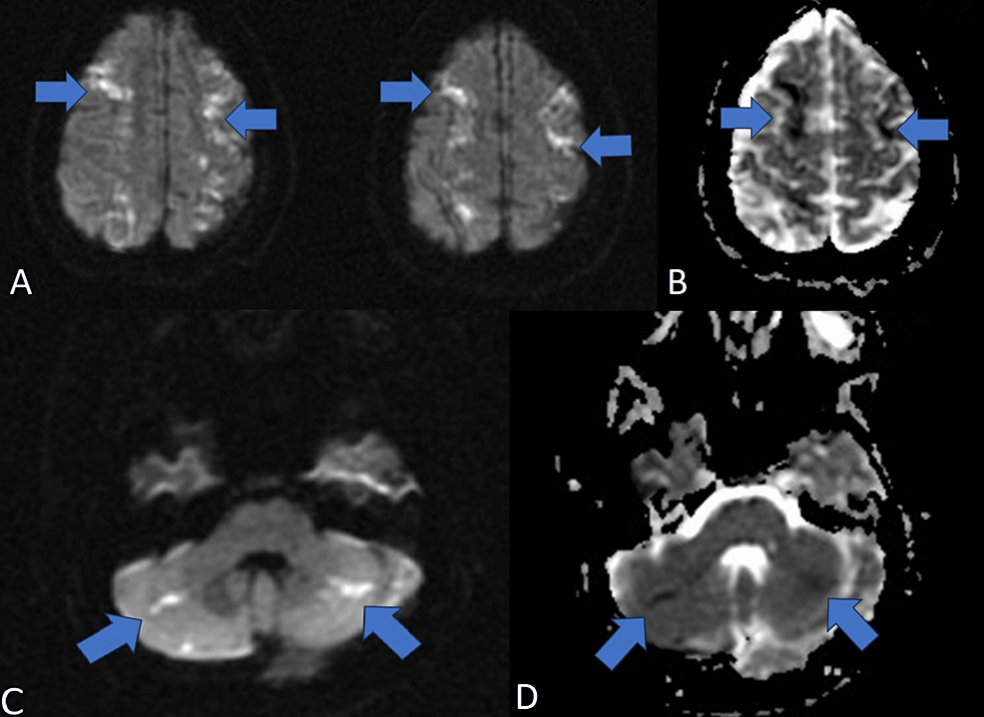
MRI head showing multiple areas of stroke suggesting thromboembolic showering (A) DWI images showcasing areas of bilateral frontal lobe infarcts (blue arrows). (B) ADC image showing areas of bilateral frontal lobe infarcts (blue arrows). (C) DWI image showing areas of bilateral cerebellar infarcts (blue arrows). (D) ADC image showing areas of bilateral cerebellar infarcts (blue arrows). DWI: diffusion-weighted imaging; ADC: apparent diffusion coefficient

She later developed a fever of 102 degrees F, so her antibiotics were escalated to vancomycin and meropenem. Blood cultures were drawn and did not grow any organisms, so infectious disease recommended discontinuing antibiotics. A hepatobiliary iminodiacetic acid (HIDA) scan with cholecystokinin was unremarkable, with no signs of acute cholecystitis. Due to persistent leukocytosis with eosinophilia despite treatment with antibiotics, Hematology was consulted, and the patient underwent a bone marrow biopsy to rule out a myeloproliferative disorder but instead revealed hypereosinophilia (Figure 4). Due to increasing concern for EM, she was placed on telemetry monitoring and cardiology was consulted. Given the potential cardiac involvement, endoscopy was deferred. Her hospital course was complicated by altered mental status, with CT head without contrast revealing a focal hypodensity in the frontal lobe, suggesting recent ischemia (Figure 5). MRI head without contrast confirmed multiple areas of acute ischemia with involvement of both the cerebral and cerebellar hemispheres, suggesting diffuse embolic or thrombotic showering (Figure 6). Subsequent MRA head and neck were unremarkable. Tissue plasminogen activator was not administered given that she was out of the therapeutic window, but her mental status gradually recovered with no residual symptoms. Her electroencephalogram was unremarkable, and the hypercoagulable workup, including serum homocysteine, antiphospholipid syndrome antibodies, Factor V and VIII, and protein C and S levels, was unrevealing (Table 1).

Per cardiology, she was transferred to another facility for further workup with the Advanced Heart Failure service. At this point, the patient was suspected to have hypereosinophilic with cardiac, prothrombotic, and gastrointestinal manifestations. Per hematology, CT neck and chest was performed to rule out lymphadenopathy, which was unremarkable. Further workup, including serum tryptase, stool ova and parasites, JAK2, BCR-ABL, fluorescence in situ hybridization (FISH), and flow cytometry was also unremarkable (Table 1). CMR with gadolinium showed a left ventricular ejection fraction (LVEF) of approximately 30% and diffusely increased myocardial thickness. T2-weighted imaging showed diffuse subendocardial increased signal intensity throughout the mid to apical cavity in the left ventricle, suggesting myocardial edema. Post-contrast images demonstrated diffuse circumferential subendocardial late gadolinium enhancement throughout the basal to the apical cavity, most pronounced along the septal wall (Video 1).

**Video 1:** Cardiac MRI showing circumferential subendocardial late gadolinium enhancement throughout the basal to the apical left ventricle

The patient was again febrile to 101.1 F with blood pressures in the 80s/50s, persistent leukocytosis with elevated eosinophil percentage, and procalcitonin (Table 1). Given concern for sepsis, a second stool ova and parasites, and repeat blood cultures were sent, which grew no organisms. Given the patient had a recent thromboembolic stroke and persistent abdominal pain, CT angiography of the abdomen and pelvis was ordered to rule out ischemic bowel, which also came back negative. Serologies for Strongyloides, trichinellosis, filariasis, toxocariasis, schistosomiasis, aspergillosis, histoplasmosis, and HIV were also unremarkable. Cefepime and metronidazole were empirically started and steroid treatment for suspected EM was deferred. Serum IgE level was within normal limits (Table 1).

Allergy/immunology was consulted to evaluate the patient given concern for HES yet unremarkable workup and normal serum IgE levels. While there was suspicion for HES, other causes of peripheral eosinophilia had to be ruled out, including parasitic infection, malignancy, autoimmune disease, including eosinophilic granulomatosis with polyangiitis, immunodeficiency, and drug-induced eosinophilia. Trypanosoma cruzi and echinococcus species serologies were sent to complete the workup for parasitic infection, which were negative. Quantiferon-TB Gold testing was also negative, ruling out a mycobacterial etiology. Malignancy was unlikely with bone marrow biopsy only significant for eosinophilia and unremarkable CT of the head, neck, chest, abdomen, and pelvis. An autoimmune disease was unlikely, with negative antinuclear antibodies and perinuclear anti-neutrophil cytoplasmic antibodies (p-ANCA). Medication-induced eosinophilia was unlikely given that she was not taking any home medications prior to admission. T-cell subsets and quantitative immunoglobulin titers were also unremarkable, effectively ruling out immunodeficiency as a potential etiology.

With workup so far unrevealing and persistent duodenitis and jejunitis seen on CT abdomen and pelvis, the patient underwent a small bowel enteroscopy with gastroenterology. This revealed congested, erythematous, and linearly eroded mucosa in the stomach as well as erythematous duodenopathy in the first part of the duodenum. Biopsies of the stomach, duodenum, and jejunum were taken, with pathology remarkable for gastric antral and oxyntic mucosa with chronic inflammation and otherwise normal small bowel. There were no signs of eosinophilia noted in the pathology of the acquired specimens.

The following day, the patient suffered a cardiac arrest secondary to ventricular fibrillation. Return of spontaneous circulation (ROSC) was achieved after approximately 14 minutes after six unsynchronized cardioversions and administration of magnesium and amiodarone. She was intubated and taken to the coronary care unit (CCU) for further management. She was started on an amiodarone infusion and later had a second episode of cardiac arrest the following day after transfer to the CCU, leading to the initiation of lidocaine infusion in addition to amiodarone infusion. She underwent an endomyocardial biopsy (EMB) of the right ventricle and was empirically started on 1 gram of intravenous methylprednisolone after the biopsies were sent for pathology given her high risk of decompensation.

**Figure 7:**
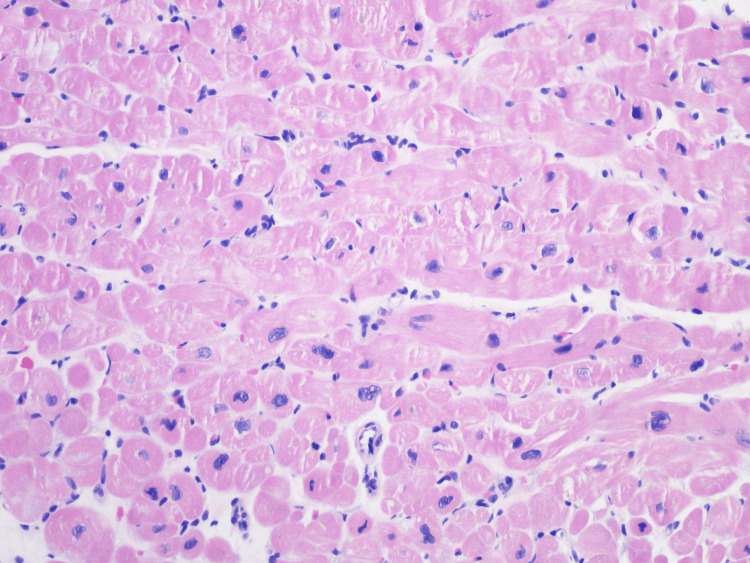
Endomyocardial biopsy H&E staining of endomyocardial biopsy at 200X magnification showing only myocyte hypertrophic changes with no evidence of inflammation

EMB pathology was remarkable only for concentric hypertrophy with no signs of inflammation or eosinophilia (Figure 7). Her leukocytosis and eosinophil counts improved after steroid initiation (Table 1). Initial TTE upon transfer to the CCU was remarkable for an LVEF of approximately 30% with global left ventricular dysfunction (Video 2). Repeat TTE three days after the initiation of steroids showed improvement of her LVEF to approximately 40% (Video 3). She had no further events on telemetry and was taken for implantable cardioverter-defibrillator implantation with electrophysiology. She was transitioned to a prednisone taper for six weeks and later downgraded to the medicine floors with no further acute events and discharged with outpatient follow-up.

**Video 2:** Transthoracic echocardiogram prior to steroid treatment Transthoracic echocardiogram four-chamber view showing LVEF of 30% with global left ventricular dysfunction LVEF: left ventricular ejection fraction

**Video 3:** Transthoracic echocardiogram after steroid treatment Transthoracic echocardiogram four-chamber view showing improved LVEF of 40% three days after empiric initiation of intravenous steroids LVEF: left ventricular ejection fraction

## Discussion

EM is a rare type of myocarditis and is considered a cardiac complication of hypereosinophilia. Typically, a condition causing the peripheral eosinophil count to be elevated causes eventual infiltration of the myocardium by eosinophils, which in turn release pro-inflammatory molecules such as eosinophil-derived neurotoxin, major basic protein, cationic protein, and other reactive oxygen species [1,2]. These molecules work in tandem to damage local endothelial cells and myocytes, leading to necrosis, eventually causing the formation of endomyocardial fibrosis in chronic cases. These local inflammatory changes can lead to damage to macrostructures within the heart, including the valves and papillary muscles, which can lead to regurgitation [1,3].

In patients with peripheral eosinophilia, typically 50-60% develop cardiac involvement, with eosinophilic infiltration of the myocardium lasting for several weeks [1,4]. There are several main etiologies to consider when facing hypereosinophilia, including eosinophilia secondary to infection such as helminthic or parasitic, eosinophilia secondary to medications or autoimmune disorders, and eosinophilia secondary to a myeloproliferative disorder, acute leukemia, or chronic myeloid disorder [1,2]. In our case presentation, the patient underwent extensive workup to rule out most of these etiologies. She tested negative for infectious workup, including stool ova and parasites, as well as serologies for most species of parasites and helminths. Her autoimmune workup was also negative, and her bone marrow biopsy was significant only for eosinophilia. She reported that she did not take any medications as an outpatient, although she was prescribed spironolactone approximately one year prior to presentation due to her diagnosis of nonischemic cardiomyopathy. Outpatient labs from her cardiologist were found to be unremarkable, with no leukocytosis or eosinophilia present, making medications an unlikely etiology. HES is an idiopathic disease that describes cases of peripheral eosinophilia after all other known etiologies have been ruled out, which was the case in our patient.

Idiopathic HES is typically defined by a peripheral eosinophil count of at least 1500 uL over six months without a known etiology, with EM as a common complication in these patients, manifesting in over 50% of patients diagnosed with idiopathic HES [1,3]. EM is considered to be the main cause of mortality in patients with HES, as myocyte necrosis is extremely common with subsequent endomyocardial scarring that predisposes patients to develop restrictive cardiomyopathy [4]. While our patient did not exhibit restrictive cardiomyopathy on imaging; this may have been due to the time course of her disease and her atypical presentation. Her previous diagnosis of nonischemic cardiomyopathy may have also affected the structural remodeling of her heart, which was initially attributed to viral myocarditis. Her EMB also did not show signs of myocardial necrosis or endomyocardial fibrosis, but this can be explained by focal infiltration and the low sensitivity of EMB. Common manifestations of EM secondary to HES include fatal arrhythmias, which our patient did suffer twice [1,4]. This may be explained by inflammation of the myocardium and scar tissue formation, forming niduses for the formation of arrhythmias. Some studies have also reported embolic events as a rare complication of EM secondary to HES, with 4% of HES patients developing embolic events in one prospective study [3]. This is mainly due to local endothelial damage and the release of major basic proteins by eosinophils, which can stimulate platelets and create a prothrombotic environment. This can predispose patients to develop myocardial infarction, but in our case, our patient did show evidence of multiple strokes in her bilateral frontal and cerebellar lobes. This was suspected to be secondary to thromboembolic showering and can be attributed to the formation of microthrombi during the initial stages of EM. Neurological involvement in HES is typically considered to be a poor prognostic indicator, making this case unique, as our patient managed to make a significant recovery [1]. HES typically involves multiple organ systems, with the most common being cutaneous manifestations, including maculopapular or pustular rashes [1]. Gastrointestinal involvement is also common, as seen in our patient, however, biopsies of her stomach and intestines showed no signs of eosinophilia. Eosinophils tend to stay in the infiltrated tissue for several weeks, and both these biopsies and the EMB were performed several weeks into her hospital course due to her clinical instability, which may have been enough time for the affected organs to be cleared. This may also be an atypical presentation of HES with shortened time courses of eosinophilic infiltration.

EM can be extremely difficult to diagnose given the clinical ambiguity of its presenting symptoms. Many patients can present with typical signs of congestive heart failure and may also note infectious or gastrointestinal symptoms that may hint at the etiology of the patient’s eosinophilia and subsequent development of EM. Patients may also present with cutaneous findings and recent administration of medications or viral illness that may culminate in the development of drug reactions with eosinophilia and systemic symptoms (DRESS) [1]. With this wide range of potential clinical presentations, no distinct criteria exist for the diagnosis of EM, with most diagnoses occurring at autopsy [5]. Electrocardiography may show nonspecific findings with ST-segment changes and T-wave abnormalities. Echocardiology may show a wide range of structural abnormalities depending on the extent of the disease, including restrictive, dilated, hypertrophic, and ischemic cardiomyopathies [1,4]. CMR imaging has recently emerged as a powerful noninvasive tool in aiding the diagnosis of myocarditis with guidance from the updated Lake Louise criteria in 2018 [6]. CMR cannot definitively diagnose myocarditis, as there is no information regarding the type of myocarditis and its degree of involvement, requiring the need for tissue diagnosis [1]. While EMB can provide definitive tissue diagnosis, poor yield, and high sampling error led to the preference of using CMR with clinical judgment. Joint guidelines issued by the American Heart Association, American College of Cardiology, and European Society of Cardiology recommend reserving EMB only for suspected cases of myocarditis with high associated mortality rates that respond to prompt immunosuppression, including acute necrotizing EM, giant cell myocarditis, and immune-checkpoint inhibitor-associated myocarditis [7]. New image-guided techniques using electroanatomical mapping, CMR, and positron emission tomography to guide EMB have shown promising results in increasing diagnostic yield, but these techniques are not readily available across all institutions [8].

## Conclusions

Here, we present a case of presumed chronic EM secondary to HES complicated by a cardiac arrest with subsequent improvement in cardiac function after treatment with intravenous corticosteroids. Her initial nonspecific symptoms on presentation and extensive unremarkable workup to determine the etiology of her peripheral eosinophilia made diagnosis challenging. Further workup with CMR raised suspicion for myocarditis, but EMB failed to provide a definitive diagnosis. After multiple episodes of cardiac arrest, intravenous corticosteroids were started, with subsequent TTE showing significant improvement in LVEF from 30% to 40%. This case presentation highlights the importance of clinical judgment and initiating treatment promptly to avoid poor patient outcomes. The development of specific criteria for diagnosing EM is necessary for prompt diagnosis and improved patient outcomes.

## References

[1] Sheikh H, Siddiqui M, Uddin SM, Haq A, Yaqoob U (2018). The clinicopathological profile of eosinophilic myocarditis. Cureus.

[2] Zhong Z, Yang Z, Peng Y, Wang L, Yuan X (2021). Diagnosis and treatment of eosinophilic myocarditis. J Transl Autoimmun.

[3] Ogbogu PU, Rosing DR, Horne MK 3rd (2007). Cardiovascular manifestations of hypereosinophilic syndromes. Immunol Allergy Clin North Am.

[4] Ommen SR, Seward JB, Tajik AJ (2000). Clinical and echocardiographic features of hypereosinophilic syndromes. Am J Cardiol.

[5] Getz MA, Subramanian R, Logemann T, Ballantyne F (1991). Acute necrotizing eosinophilic myocarditis as a manifestation of severe hypersensitivity myocarditis. Antemortem diagnosis and successful treatment. Ann Intern Med.

[6] Davies J, Spry CJ, Sapsford R, Olsen EG, de Perez G, Oakley CM, Goodwin JF (1983). Cardiovascular features of 11 patients with eosinophilic endomyocardial disease. Q J Med.

[7] Kiamanesh O, Toma M (2021). The state of the heart biopsy: a clinical review. CJC Open.

[8] Caforio AL, Pankuweit S, Arbustini E (2013). Current state of knowledge on aetiology, diagnosis, management, and therapy of myocarditis: a position statement of the European Society of Cardiology Working Group on Myocardial and Pericardial Diseases. Eur Heart J.

